# The COVID-19 Pandemic Period, SARS-CoV-2 Infection, and Perinatal Health

**DOI:** 10.1001/jamanetworkopen.2024.10696

**Published:** 2024-05-09

**Authors:** Shelley Jung, Emily F. Liu, Dana E. Goin, Kara E. Rudolph, Mahasin S. Mujahid, William H. Dow, Jennifer Ahern

**Affiliations:** 1 Division of Epidemiology, School of Public Health, University of California, Berkeley; 2 Department of Epidemiology, Mailman School of Public Health, Columbia University, New York, New York; 3 Division of Health Policy, School of Public Health, University of California, Berkeley

## Abstract

This cohort study assesses population-level associations of COVID-19 with birth parent and infant health, distinguishing the COVID-19 pandemic period from individual SARS-CoV-2 infection.

## Introduction

SARS-CoV-2 infection has physiologic effects on pregnancy that influence perinatal health, including immune and inflammatory responses that affect placental function.^1^ To date, connections of COVID-19 with adverse perinatal outcomes have been documented in large population studies and meta-analyses,^2^ but research separating individual SARS-CoV-2 infection from other changing risks during the pandemic is lacking. Few studies have considered associations of the COVID-19 pandemic period with perinatal health. We aimed to quantify population-level associations of COVID-19 with birth parent and infant health, distinguishing the COVID-19 pandemic period from individual SARS-CoV-2 infection, to better understand associations between the pandemic and perinatal health.

## Methods

This cohort study examines statewide California data, individually linking all birth and hospital discharge records for 2019 to 2020, subset to April to December yearly. Among individuals who delivered live infants, we compared: (1) 2020 birth parents with SARS-CoV-2 infection, (2) 2020 birth parents without SARS-CoV-2 infection, and (3) 2019 prepandemic birth parents. We examined the following outcomes: preterm birth (PTB), hypertensive disorders of pregnancy (HDP), gestational diabetes (GD), and severe maternal morbidity (SMM). We separated the association of SARS-CoV-2 infection ([1] vs [2]) from broader societal changes during the COVID-19 pandemic period ([2] vs [3]). We applied model-based standardization (g-computation)^3^ to estimate a risk difference (RD) for the amount of additional risk experienced by the exposed group (eg, group with SARS-CoV-2 infection or COVID-19 pandemic period deliveries) that is associated with the exposure, adjusted for individual (insurance, parity, education, age, race and ethnicity) and community-level confounders (racial and ethnic composition, poverty, median household income, unemployment, vehicle ownership, vacancy rates, marital status). We followed the STROBE reporting guideline. Statistical analysis was performed from December 2023 to February 2024 using R version 4.2.1 (R Project for Statistical Computing); 95% CIs were used to assess the precision of parameter estimates, and 95% CIs excluding the null were considered statistically significant. See eMethods in Supplement 1 for additional details.

## Results

Table 1 describes birth parents who delivered in 2020 with (n = 7150; 377 [5.3%] Asian; 5266 [73.7%] Hispanic; 1021 [14.3%] White; mean [SD] age, 28.45 [5.99] years) and without (n = 270 872; 39 020 [14.4%] Asian; 131 634 [48.6%] Hispanic; 77 055 [28.4%] White; mean [SD] age, 30.27 [5.79] years) SARS-CoV-2 infection and in 2019 (n = 300 030; 47 385 [15.8%] Asian; 144 844 [48.3%] Hispanic; 82 935 [27.6%] White; mean [SD] age: 30.12 [5.82] years). Birth parents with SARS-CoV2 infection were more likely to be Hispanic, have lower education, receive public insurance, and live in lower income neighborhoods compared with the other groups. Table 2 shows the adjusted associations of SARS-CoV-2 infection. There were higher burdens of PTB (RD, 2.8% [95% CI, 2.1%-3.5%]), HDP (RD, 3.3% [95% CI, 2.4%-4.1%]), and SMM (RD, 2.3% [95% CI, 1.9%-2.7%]) among birth parents with SARS-CoV-2 infection (vs 2020 without infection). Table 2 shows the adjusted associations of the COVID-19 pandemic period. There was a lower burden of PTB (RD, −0.4% [−0.6% to −0.3%]), particularly spontaneous PTB, and a higher burden of HDP (RD, 1.1% [95% CI, 0.9% to 1.2%]) and GD (RD, 0.9% [0.8%-1.1%]) among 2020 birth parents without SARS-CoV-2 infection (vs 2019). Overall, SARS-CoV-2 infection was associated with adverse perinatal health. The pandemic period was associated with decreased risk of PTB and increased risk of birth parent conditions.

**Table 1.** Characteristics of Birth Parents by SARS-CoV-2 Infection and Year

	COVID-19 pandemic period (2020)		Prepandemic (2019)
	With SARS-CoV-2 infection^a^	Without SARS-CoV-2 infection	
Births, No.^b^	7150	270 872	300 030
Perinatal health outcomes, No. (%)^c^			
Preterm birth	832 (11.6)	22 935 (8.5)	26 560 (8.9)
Spontaneous	603 (8.4)	16 491 (6.1)	19 351 (6.4)
Indicated	229 (3.2)	6444 (2.4)	7202 (2.4)
Hypertensive disorders of pregnancy	1106 (15.5)	34 594 (12.8)	34 490 (11.5)
Gestational diabetes	864 (12.1)	32 798 (12.1)	33 089 (11.0)
Severe maternal morbidity	236 (3.3)	2707 (1.0)	2997 (1.0)
Birth parent characteristics^d^			
Age, mean (SD), y	28.45 (5.99)	30.27 (5.79)	30.12 (5.82)
Race and ethnicity, No. (%)^e^			
American Indian or Alaska Native	17 (0.2)	900 (0.3)	966 (0.3)
Asian	377 (5.3)	39 020 (14.4)	47 385 (15.8)
Black	318 (4.4)	13 951 (5.2)	14 996 (5.0)
Hawaiian or Pacific Islander	35 (0.5)	1042 (0.4)	1164 (0.4)
Hispanic	5266 (73.7)	131 634 (48.6)	144 844 (48.3)
Multiracial	108 (1.5)	6997 (2.6)	7401 (2.5)
White	1021 (14.3)	77 055 (28.4)	82 935 (27.6)
Other race	8 (0.1)	273 (0.1)	339 (0.1)
Insurance type, No. (%)			
Other	12 (0.2)	1003 (0.4)	1387 (0.5)
Private coverage	2248 (31.4)	144 481 (53.3)	153 246 (51.1)
Public coverage	4790 (67.0)	121 927 (45.0)	135 581 (45.2)
Self-pay	100 (1.4)	3461 (1.3)	9816 (3.3)
Parity, No. (%)			
0	2474 (34.6)	107 422 (39.7)	118 804 (39.6)
1	2040 (28.5)	86 150 (31.8)	96 398 (32.1)
≥2	2636 (36.9)	77 300 (28.5)	84 828 (28.3)
Education, No. (%)			
College graduation or more	1509 (21.1)	116 916 (43.2)	127 055 (42.3)
Less than college	4117 (57.6)	123 442 (45.6)	136 722 (45.6)
Less than high school	1524 (21.3)	30 514 (11.3)	36253 (12.1)
Community characteristics, mean (SD)^f^			
% Asian	9.7 (10.4)	13.3 (13.8)	13.76 (14.4)
% Black	6.5 (7.1)	5.9 (7.3)	5.88 (7.3)
% Hispanic	54.5 (23.2)	42.8 (24.4)	42.94 (24.5)
% Below poverty line	16.5 (7.8)	14.3 (7.9)	14.34 (7.9)
Median household income, $	66 148 (22 746)	76 346 (30 598)	76 140 (30 342)
% On public assistance	4.5 (2.8)	3.8 (2.8)	3.8 (2.8)
% With 0 vehicles	6.9 (5.5)	6.8 (5.6)	6.8 (5.7)
Owner vacancy rate	1.2 (1.2)	1.1 (1.2)	1.1 (1.2)
Renter vacancy rate	3.2 (2.0)	3.4 (2.3)	3.4 (2.3)
% Unemployed	7.3 (3.0)	6.5 (2.9)	6.5 (2.9)
% Never married	39.1 (7.3)	37.4 (8.1)	37.4 (8.1)
% Separated	2.4 (0.8)	2.1 (0.9)	2.1 (0.9)

^a^ SARS-CoV-2 infection at delivery defined based on *International Statistical Classification of Diseases and Related Health Problems, Tenth Revision* codes in the inpatient hospital discharge record and complications of pregnancy in the birth record (eMethods in Supplement 1).

^b^ Includes all birth records April-December 2019 and April-December 2020 with complete covariate data (8.3% [2019] and 8.4% [2020] excluded due to missing covariates) (eMethods in Supplement 1).

^c^ Perinatal health outcomes are derived from both the vital statistics birth record and the hospital discharge records during the pregnancy or at delivery (eMethods in Supplement 1).

^d^ Individual characteristics for the birth parent at delivery are from the vital statistics birth record (birth parent’s age, race and ethnicity, parity, education) and from the delivery hospital discharge record (insurance type) (eMethods in Supplement 1).

^e^ Race and ethnicity for the birth parent was self-reported on the birth certificate according to the classifications used by the California Department of Public Health. Up to 3 race categories may be selected, in addition to Hispanic ethnicity. The other race category is provided as an option for the birth parent to select when self-reporting their race and ethnicity. They may select up to 3 race categories, in addition to Hispanic ethnicity.

^f^ Community characteristics for the zip code tabulation area of birth parent’s residence at the time of delivery from the American Community Survey 2019 5-Year Estimates (eMethods in Supplement 1).

**Table 2.** Adjusted Marginal Risk Differences for SARS-CoV-2 Infection and the COVID-19 Pandemic Period^a^

Outcome	R1^b^	R0^c^	RD (95% CI)^d^
**COVID-19 pandemic period with SARS-CoV-2 infection (2020, n = 7150) vs COVID-19 pandemic period without SARS-CoV-2 infection (2020, n = 270 872)**			
Preterm birth	0.116	0.089	0.028 (0.021 to 0.035)
Spontaneous	0.084	0.064	0.020 (0.014 to 0.027)
Indicated	0.032	0.025	0.007 (0.004 to 0.011)
Hypertensive disorders of pregnancy	0.155	0.122	0.033 (0.024 to 0.041)
Gestational diabetes	0.121	0.114	0.007 (0.000 to 0.015)
Severe maternal morbidity	0.033	0.010	0.023 (0.019 to 0.027)
**COVID-19 pandemic period without SARS-CoV-2 infection (2020, n = 270 872) vs prepandemic (2019, n = 300 030)**			
Preterm birth	0.085	0.089	−0.004 (−0.006 to −0.003)
Spontaneous	0.061	0.065	−0.004 (−0.005 to −0.003)
Indicated	0.024	0.024	0.000 (−0.001 to 0.000)
Hypertensive disorders of pregnancy	0.128	0.117	0.011 (0.009 to 0.012)
Gestational diabetes	0.121	0.112	0.009 (0.008 to 0.011)
Severe maternal morbidity	0.010	0.010	0.000 (−0.001 to 0.000)

^a^ Adjusted for birth parent’s age, race and ethnicity, insurance type, parity, education, and community characteristics (full list of variables and their categorization in Table 1). Parameters estimated with model-based standardization (g-computation) (eMethods in Supplement 1). Estimation with matching (generalized full matching) and targeted maximum likelihood estimation (TMLE) resulted in essentially equivalent results.

^b^ R1 is the estimated risk of the outcome if the exposed group (eg, group with SARS-CoV-2 infection) experienced the exposure, adjusted for covariates.

^c^ R0 is the estimated risk of the outcome if the exposed group had not experienced the exposure, adjusted for covariates.

^d^ RD is the risk difference R1 − R0, which estimates the amount of additional risk experienced by the exposed group (eg, group with SARS-CoV-2 infection, or COVID-19 pandemic period deliveries) that is associated with the exposure.

## Discussion

This study adds to understanding of the associations between COVID-19 and perinatal health in a large, diverse population by distinguishing the connections of SARS-CoV-2 infection from those of the COVID-19 pandemic period with PTB and birth parent conditions. Our findings suggest that SARS-CoV-2 infection was associated with increased PTB, HDP, and SMM, consistent with other studies.^2,4^ Interestingly, the pandemic period was associated with decreased risk of PTB, but increased risk of HDP and GD; this has been reported elsewhere, but not using designs that could disentangle infection from the pandemic period.^5,6^ The pandemic period association with decreased risk of PTB was largest for spontaneous PTB, suggesting that it may be explained by socioenvironmental and behavioral modifications, such as commute changes and reduced non-SARS-CoV-2 infections. Increased risk of HDP and GD may be due to stress and reduced and/or remote prenatal care due to the pandemic. Limitations include SARS-CoV-2 infection identification at delivery and temporality for some comparisons (see eMethods in Supplement 1). Our study adds to the literature by distinguishing individual SARS-CoV-2 infection from the COVID-19 pandemic period in connection with perinatal outcomes.

## Notes

Supplement 1.
eMethods.

Supplement 2.
Data Sharing Statement

## References

[1] Cavalcante MB, Cavalcante CTMB, Sarno M, Barini R, Kwak-Kim J. Maternal immune responses and obstetrical outcomes of pregnant women with COVID-19 and possible health risks of offspring. J Reprod Immunol. 2021;143:103250. doi:10.1016/j.jri.2020.103250 33249335 PMC7676367

[2] Marchand G, Patil AS, Masoud AT, . Systematic review and meta-analysis of COVID-19 maternal and neonatal clinical features and pregnancy outcomes up to June 3, 2021. AJOG Glob Rep. 2022;2(1):100049. doi:10.1016/j.xagr.2021.100049 35005663 PMC8720679

[3] Ahern J, Hubbard A, Galea S. Estimating the effects of potential public health interventions on population disease burden: a step-by-step illustration of causal inference methods. Am J Epidemiol. 2009;169(9):1140-1147. doi:10.1093/aje/kwp015 19270051 PMC2732980

[4] Ferrara A, Hedderson MM, Zhu Y, . Perinatal complications in individuals in California with or without SARS-CoV-2 infection during pregnancy. JAMA Intern Med. 2022;182(5):503-512. doi:10.1001/jamainternmed.2022.0330 35311909 PMC8938896

[5] Molina RL, Tsai TC, Dai D, . Comparison of pregnancy and birth outcomes before vs during the COVID-19 pandemic. JAMA Netw Open. 2022;5(8):e2226531. doi:10.1001/jamanetworkopen.2022.26531 35960517 PMC9375166

[6] Chmielewska B, Barratt I, Townsend R, . Effects of the COVID-19 pandemic on maternal and perinatal outcomes: a systematic review and meta-analysis. Lancet Glob Health. 2021;9(6):e759-e772. doi:10.1016/S2214-109X(21)00079-6 33811827 PMC8012052

