# Supplementary material for: The COVID-19 Pandemic Period, SARS-CoV-2 Infection, and Perinatal Health
Source: JAMA Netw Open. 2024 May 9;7(5):e2410696. doi: 10.1001/jamanetworkopen.2024.10696 (PMC11082682; doi:10.1001/jamanetworkopen.2024.10696)
Supplement: Supplement 1. — eMethods. [file jamanetwopen-e2410696-s001.pdf]

Supplemental Online Content

Jung S, Liu EF, Goin DE, et al. The COVID-19 pandemic period, SARS-CoV-2 infection, and perinatal health. *JAMA Netw Open.* 2024;7(5):e2410696. doi:10.1001/jamanetworkopen.2024.10696

This supplemental material has been provided by the authors to give readers additional information about their work.

© 2024 Jung S et al. *JAMA Network Open.*

eMethods

1. Ethical Review of Study
2. Data Linkage
3. Exposure
4. Outcomes
5. Covariates
6. Analysis
7. Limitations

1. Ethical Review of Study

This study was reviewed and approved by the California Health and Human Services Agency and the University of California, Berkeley Committees for the Protection of Human Subjects. The study involved secondary analysis of surveillance data from California state agencies, and participant consent was waived.

2. Data Linkage

To create the linked data set, we worked with the 2019 Birth Cohort File and 2020 Comprehensive Birth File (released earlier than the Birth Cohort File) from the California Department of Public Health, Center for Health Statistics and Informatics, and the 2019 and 2020 inpatient hospital discharge data (Patient Discharge Data PDD) from the California Department of Health Care Access and Information. The primary approach was to link all live births with birth parent hospital records on the hospital identification code and the birth parent's date of birth. If a match was not identified, birth parent's admit date, baby's date of birth, and birth parent's ZIP code of residence were used to match. If a match was still not identified, birth parent's date of birth and ZIP code of residence were used to match. Using the primary linkage approach, 96.4% and 96.2% of hospital births were matched in 2019 and 2020, respectively. Overall (after the primary and secondary approaches), 97.3% and 97.4% of hospital births were matched in 2019 and 2020, respectively.

3. Exposure

Birth parents were considered to have SARS-CoV-2 infection if the inpatient hospital discharge record included the ICD10 code U071 for COVID-19 (introduced in April 2020), or if the birth record included the "Complications and procedures of pregnancy and concurrent illness" codes 91 and 92 for presumed and confirmed COVID-19, respectively (introduced in June 2020). Birth parents were considered exposed to the COVID-19 pandemic period only if they delivered in April-December 2020 and did not have a record of SARS-CoV-2 infection. Birth parents who delivered in April-December 2019 were unexposed to SARS-CoV-2 infection and the COVID-19 pandemic period. Recent research on California deliveries during the pandemic ascertained that about 85% of births were in facilities with confirmed universal testing, and that associations between SARS-CoV-2 infection and outcomes were very similar when restricting to the universal testing subset of deliveries (1).

4. Outcomes

Perinatal health outcomes were classified based on combinations of the birth record codes and hospitalization ICD10 codes from pregnancy or delivery, as specified in eTable 1. Where ICD10 codes were used, the specific codes for each outcome are listed in eTable 2.

eTable 1. Definitions of perinatal health outcomes

OUTCOME	DEFINITION
**Preterm birth (PTB)**	Gestational weeks at birth less than 37 (birth record)
**Spontaneous (2)**	Preterm premature rupture of membranes (PPROM) (ICD10 or birth record)
	Vaginal delivery without PPROM (ICD10 or birth record)
**Indicated (2)**	Preterm induction of labor (IOL) without PPROM (ICD10 or birth record)

© 2024 Jung S et al. *JAMA Network Open.*

	Cesarean delivery without IOL or PPROM (ICD10 or birth record)
**Hypertensive disorder of pregnancy (HDP) (3)**	Preeclampsia (ICD10 code or birth record)<br>Eclampsia (ICD10 code or birth record)<br>Hypertension during pregnancy (ICD10 or birth record)<br>No record of prior hypertension (ICD10 or birth record)
**Gestational diabetes (GD) (4)**	Gestational diabetes (ICD10 code or birth record)<br>No record of prior diabetes (ICD10 code or birth record)
**Severe maternal morbidity (SMM) (5)**	SMM (ICD10 codes [without transfusion])

eTable 2. ICD10 codes from the hospitalization records used to identify elements of perinatal outcomes

CONDITION	ICD10 CODE
Preterm premature rupture of membranes (2)	O42011, O42012, O42013, O42019, O42111, O42112, O42113, O42119, O42911, O42912, O42913, O42919, P011
Preeclampsia (3)	O11, O14
Eclampsia (3)	O15
Hypertension during pregnancy (3)	O10, O11, O13, O14, O15, O16
Pre-pregnancy hypertension (3)	I10, I11, I12, I13, I15
Gestational diabetes (4)	O244, O249, O9981
Diabetes (4)	E08, E09, E10, E11, E13, O240, O241, O243, O248

© 2024 Jung S et al. *JAMA Network Open.*

SMM (diagnosis codes) (5)	I2101, I2102, I2109, I2111, I2119, I2121, I2129, I213, I214, I219, I21A1, I21A9, I220, I221, I222, I228, I229, I7100, I7101, I7102, I7103, I711, I712, I713, I714, I715, I716, I718, I719, I790, N170, N171, N172, N178, N179, O904, J80, J951, J952, J953, J95821, J95822, J9600, J9601, J9602, J9620, J9621, J9622, R092, O88111, O88112, O88113, O8812, O8813, I462, I468, I469, I4901, I4902, D65, D688, D689, O723, O1500, O1502, O1503, O151, O152, O159, O1422, O1423, I97120, I97121, I97130, I97131, I97710, I97711, I6000, I6001, I6002, I6010, I6011, I6012, I602, I6030, I6031, I6032, I604, I6050, I6051, I6052, I606, I607, I608, I609, I611, I612, I613, I614, I615, I616, I618, I619, I6200, I6201, I6202, I6203, I621, I629, I6300, I63011, I63012, I63013, I63019, I6302, I63031, I63032, I63033, I63039, I6309, I6310, I63111, I63112, I63113, I63119, I6312, I63131, I63132, I63133, I63139, I6319, I63211, I63212, I63213, I63219, I6322, I63231, I63232, I63233, I63239, I6329, I6330, I63311, I63312, I63313, I63319, I63321, I63322, I63323, I63329, I63331, I63332, I63333, I63339, I63341, I63342, I63343, I63349, I6339, I6340, I63411, I63412, I63413, I63419, I63421, I63422, I63423, I63429, I63431, I63432, I63433, I63439, I63441, I63442, I63443, I63449, I6349, I6350, I63511, I63512, I63513, I63519, I63521, I63522, I63523, I63529, I63531, I63532, I63533, I63539, I63541, I63542, I63543, I63549, I6359, I636, I6381, I6389, I639, I6501, I6502, I6503, I6509, I651, I6521, I6522, I6523, I6529, I658, I659, I6601, I6602, I6603, I6609, I6611, I6612, I6613, I6619, I6621, I6622, I6623, I6629, I663, I668, I669, I670, I671, I672, I673, I674, I675, I676, I677, I6781, I6782, I6783, I67841, I67848, I67850, I67858, I6789, I679, I680, I682, I688, O2251, O2252, O2253, I97810, I97811, I97820, I97821, O873, J810, I501, I5020, I5021, I5023, I5030, I5031, I5033, I5040, I5041, I5043, I509, O740, O741, O742, O743, O8901, O8909, O891, O892, O85, O8604, T80211A, T8140XA, T8141XA, T8142XA, T8143XA, T8144XA, T8149XA, T8144, T8144XD, T8144XS, R6520, A400, A401, A403, A408, A409, A4101, A4102, A411, A412, A413, A414, A4150, A4151, A4152, A4153, A4159, A4181, A4189, A419, A327, O751, R570, R571, R578, R579, R6521, T782XXA, T882XXA, T886XXA, T8110XA, T8111XA, T8119XA, D5700, D5701, D5702, D57211, D57212, D57219, D57411, D57412, D57419, D57811, D57812, D57819, I2601, I2602, I2609, I2690, I2692, I2699, O88011, O88012, O88013, O88019, O8802, O8803, O88211, O88212, O88213, O88219, O8822, O8823, O88311, O88312, O88313, O88319, O8832, O8833, O8881, O8882, O8883
SMM (procedure codes) (5)	5A2204Z, 5A12012, 0UT90ZZ, 0UT94ZZ, 0UT97ZZ, 0UT98ZZ, 0UT9FZZ, 0B110Z4, 0B110F4, 0B113Z4, 0B113F4, 0B114Z4, 0B114F4, 5A1935Z, 5A1945Z, 5A1955Z

5. Covariates

We adjusted for individual- and community-level demographic and socioeconomic characteristics that may confound the relation between SARS-CoV-2 infection or the COVID-19 pandemic period and the health outcomes of birth parents and infants. Individual characteristics for the birth parent at delivery are from the vital statistics birth record (birth parent's age, race and ethnicity, parity, education) and from the delivery hospital discharge record (insurance type). Community characteristics for the zip code tabulation area of the birth parent's residence at the time of delivery are from the American Community Survey 2019 5-Year Estimates. For SARS-CoV-2 infection, it is well documented that particular racial and ethnic, socioeconomic, and age groups were at higher risk of SARS-CoV-2 infection due to jobs that could not be done remotely, increased crowding, and numerous other vulnerabilities to infection (6). Furthermore, community characteristics that capture structural inequities (such as segregation), or manifestations of those inequities, have been documented to increase the risk of SARS-CoV-2 infection beyond individual risk factors (6). These individual and community characteristics also have well established connections with gestational health (7,8). For the COVID-19 pandemic period, there is less concern about confounding because most 2020 deliveries were conceived before the pandemic, but control for these individual and community characteristics is a conservative approach to account for any small differences between the years.

6. Analysis

We used model-based standardization, also known as g-computation, for the analysis (9). First, a regression model (logistic regression) was used to estimate the relation between the exposure (SARS-CoV-2 infection or the COVID-19 pandemic period) and outcome (each of the perinatal health outcomes), adjusting for confounders. Next, that model was used to impute the outcome for each observation under different exposures. In this application, we

© 2024 Jung S et al. *JAMA Network Open.*

imputed outcomes while setting exposure to 1 (exposure to SARS-CoV-2 infection or exposure to the COVID-19 pandemic period), and then imputed outcomes while setting exposure to 0 (no SARS-CoV-2 infection or no COVID-19 pandemic period exposure). The imputed values were averaged across the exposed group for each analysis to estimate the 'average effect of treatment on the treated' (ATT). We took the difference between two averaged risks to estimate the ATT: R1 - the estimated risk of the outcome if the exposed group (e.g., SARS-CoV-2 infection) experienced the exposure, adjusted for covariates, and R0 - the estimated risk of the outcome if the exposed group had not experienced the exposure, adjusted for covariates. ATT (RD) is the risk difference R1-R0, which estimates the amount of additional risk experienced by the exposed group (the group exposed to SARS-CoV-2 infection or the group exposed to the COVID-19 pandemic period) that is associated with the exposure. Finally, we used the percentile method to construct 95% confidence intervals from 500 bootstrapped replications. We performed all analyses in January 2024 using R version 4.2.1. We manually coded the g-computation analysis and used the `boot` package for the bootstrapping.

We focused on the ATT as the parameter of interest because it specified the most interpretable research question or hypothetical experiment. In the examination of birth parents with SARS-CoV-2 infection, the ATT estimates how their outcomes would have been different had they not experienced the infection. For examination of the COVID-19 pandemic period, the ATT estimates how birth parents delivering in 2020 would have had different outcomes had they instead delivered pre-pandemic.

7. Limitations

This study has several limitations. SARS-CoV-2 infection was assessed at delivery because most California hospitals were testing universally at delivery (about 85% were confirmed to be universally testing). However, a lack of information on SARS-CoV-2 infections during pregnancy, but prior to delivery, means we are missing some infections, introducing some misclassification of the exposure. We anticipate that some in the 2020 no infection group should have been classified as infected. Due to measuring SARS-CoV-2 only at delivery, we do not have appropriate temporal ordering between the infection and the pregnancy complications that develop over the course of pregnancy, specifically HDP and GD. We also lacked information on the severity of SARS-CoV-2 infection, which may be relevant to perinatal outcomes. Health care access during the COVID-19 pandemic period was likely to have been impacted in novel ways due to variations in how health systems adapted to the pandemic conditions, which procedures were delayed during different time periods, and how video and other remote health services were or were not incorporated. Although we were unable to assess differential health care access directly, we controlled for the individual variable of insurance type from the hospital discharge record, which was the strongest proxy available. While we examined a range of important birth parent health outcomes, we were limited to those that can be assessed validly using hospital and birth certificate records. All individual covariates derived from the vital statistics birth record and from the delivery hospital discharge record are highly complete, but there may be some misclassification. There is also the possibility of selection bias due to the 8% of records that were omitted due to missing information needed to create study variables.

References

1. Torche F, Nobles J. Vaccination, immunity, and the changing impact of COVID-19 on infant health. *Proceedings of the National Academy of Sciences*. 2023;120(49):e2311573120. doi:10.1073/pnas.2311573120

2. Klebanoff MA, Yossef-Salameh L, Latimer C, et al. Development and Validation of an Algorithm to Determine Spontaneous versus Provider-Initiated Preterm Birth in US Vital Records. *Paediatric Perinatal Epid*. 2016;30(2):134-140. doi:10.1111/ppe.12267

3. Ford ND, Cox S, Ko JY, et al. Hypertensive Disorders in Pregnancy and Mortality at Delivery Hospitalization — United States, 2017–2019. *MMWR Morb Mortal Wkly Rep*. 2022;71(17):585-591. doi:10.15585/mmwr.mm7117a1

4. Hsu S, Selen DJ, James K, et al. Assessment of the Validity of Administrative Data for Gestational Diabetes Ascertainment. *American Journal of Obstetrics & Gynecology MFM*. 2023;5(2):100814. doi:10.1016/j.ajogmf.2022.100814

5. CDC. How Does CDC Identify Severe Maternal Morbidity? Published July 3, 2023. Accessed January 3, 2024. https://www.cdc.gov/reproductivehealth/maternalinfanthealth/smm/severe-morbidity-ICD.htm

© 2024 Jung S et al. *JAMA Network Open.*

6. Rozenfeld Y, Beam J, Maier H, et al. A model of disparities: risk factors associated with COVID-19 infection. *International Journal for Equity in Health*. 2020;19(1):126. doi:10.1186/s12939-020-01242-z

7. Goldenberg RL, Culhane JF, Iams JD, Romero R. Epidemiology and causes of preterm birth. *The Lancet*. 2008;371(9606):75-84. doi:10.1016/S0140-6736(08)60074-4

8. Wang E, Glazer KB, Howell EA, Janevic TM. Social Determinants of Pregnancy-Related Mortality and Morbidity in the United States: A Systematic Review. *Obstet Gynecol*. 2020;135(4):896-915. doi:10.1097/AOG.0000000000003762

9. Ahern J, Hubbard A, Galea S. Estimating the Effects of Potential Public Health Interventions on Population Disease Burden: A Step-by-Step Illustration of Causal Inference Methods. *American Journal of Epidemiology*. 2009;169(9):1140-1147. doi:10.1093/aje/kwp015

© 2024 Jung S et al. *JAMA Network Open.*
