# Supplementary material for: The COVID-19 Pandemic Period, SARS-CoV-2 Infection, and Perinatal Health
Source: JAMA Netw Open. 2024 May 9;7(5):e2410696. doi: 10.1001/jamanetworkopen.2024.10696 (PMC11082682; doi:10.1001/jamanetworkopen.2024.10696)
Supplement: Supplement 2. — Data Sharing Statement [file jamanetwopen-e2410696-s002.pdf]

Data Sharing Statement

Jung. The COVID-19 Pandemic Period, SARS-CoV-2 Infection, and Perinatal Health. *JAMA Netw Open*. Published May 09, 2024. doi:10.1001/jamanetworkopen.2024.10696

Data
Data available: No

Additional Information
Explanation for why data not available: Birth and hospital discharge data are available upon request for research projects from the California Department of Public Health, Center for Health Statistics and Informatics, and from the California Department of Health Care Access and Information.
